# Crosslinked Polyesters as Fully Biobased Coatings with Cutin Monomer from Tomato Peel Wastes

**DOI:** 10.3390/polym16050682

**Published:** 2024-03-02

**Authors:** Eleonora Ruffini, Andrea Bianchi Oltolini, Mirko Magni, Giangiacomo Beretta, Marco Cavallaro, Raffaella Suriano, Stefano Turri

**Affiliations:** 1 Department of Chemistry, Materials and Chemical Engineering “Giulio Natta”, Politecnico di Milano, Piazza Leonardo da Vinci 32, 20133 Milano, Italy; eleonora.ruffini@polimi.it (E.R.); marco.cavallaro@polimi.it (M.C.); stefano.turri@polimi.it (S.T.); 2 Department of Environmental Science and Policy, Università degli Studi di Milano, Via Celoria 2, 20133 Milano, Italy; mirko.magni@unimi.it (M.M.); giangiacomo.beretta@unimi.it (G.B.)

**Keywords:** cutin, agro-waste, polyester resins, coatings, 2,5-furandicarboxylic acid

## Abstract

Cutin, one of the main structural components of tomato peels, is a waxy biopolymer rich in hydroxylated fatty acids. In this study, 10,16-dihydroxyhexadecanoic acid (10,16-diHHDA) was extracted and isolated from tomato peels and exploited to develop fully crosslinked polyesters as potential candidates for replacing fossil-based metal protective coatings. A preliminary screening was conducted to select the base formulation, and then a design of experiments (DoE) was used as a methodology to identify the optimal composition to develop a suitable coating material. Different formulations containing 10,16-diHHDA and other biorefinery monomers, including 2,5-furandicarboxylic acid, were considered. To this end, all polyesters were characterized through differential scanning calorimetry (DSC) and gel content measurements to determine their T_g_ value and crosslinking efficiency. Compositions exhibiting the best trade-off between T_g_ value, chemical resistance, and sufficiently high 10,16-diHHDA content between 39 and 48 wt.% were used to prepare model coatings that were characterized for assessing their wettability, scratch hardness, chemical resistance, and adhesion to metal substrates. These polyester coatings showed a T_g_ in the range of 45–55 °C, a hydrophobic behavior with a water contact angle of around 100°, a good solvent resistance (>100 MEK double rubs), and an adhesion strength to steel higher than 2 MPa. The results obtained confirmed the potential of cutin-based resins as coatings for metal protection, meeting the requirements for ensuring physicochemical properties of the final product, as well as for optimizing the valorization of such an abundant agri-food waste as tomato peels.

## 1. Introduction

Cutin is a polyfunctional biopolyester constituted of C16 and C18 fatty acids, with dihydroxylated C16 fatty acids being the most abundant ones (more than 60 wt.%) [1,2,3,4]. Cutin is one of the main constituents (between 40 and 80 wt.%) of the plant cuticle, the external layer covering and protecting the aerial parts of plants [5,6,7]. Therefore, it is extensively available and easily recoverable from different agricultural sources, among which tomato peel is the best option [8,9,10].

Tomato is the second vegetable source produced and consumed worldwide, next to potatoes. Global tomato production is estimated at around 160 Mt/y, of which up to 40% are processed (i.e., 40 Mt/y), with California, China, and some UE countries (Italy, Spain, and Portugal) being major players [11]. A quantity of around 5–30% of tomato pomace is normally lost as food waste and is used as animal feed or disposed of in landfills [12]. Instead, it may become an important biosource of sustainable chemicals and monomers. About 27% of that tomato pomace is represented by skin, of which cutin is a major component (40–80% by weight), leading to an estimated global production potential of 0.2–2.5 Mt/y of cutin [13,14].

Based on these data, cutin is gaining interest as a viable alternative to petroleum-based monomers and polymers in some target applications. Indeed, thanks to its remarkable properties, cutin has been considered as a promising candidate for the development of plant cuticle-like biobased materials to be employed in the food packaging sector, in line with circular bioeconomy principles and guidelines [15,16,17].

From this point of view, biodegradability is one of the main features that make cutin appealing, since it can be decomposed in soil in a reasonably short time (i.e., three to eight months) at a similar rate compared to bacterial polyhydroxyalkanoates and cellulose, exhibiting higher degradation efficiency than polylactic acid [18,19,20,21]. As regards mechanical properties, cutin polymers show higher elongation at break compared to other commercial plastics and bioplastics, despite being less rigid [22]. Indeed, tomato cutin exhibits a mechanical behavior similar to that of some elastomers, with an elongation at break of 27% and Young’s modulus of 45 MPa at 23 °C and 40% relative humidity, making it more ductile and less rigid than other polymers such as PLA, P3HB, and cellulose [23]. Furthermore, cutin’s non-toxicity and water resistance make it a particularly suitable material for metal packaging containers like cans, where food contact approval, chemical inertness, and mechanical robustness are essential requirements that must be ensured [24,25]. Innovative solutions for converting tomato skin into biodegradable plastic (BIOPROTO EU-funded project) [26] and green lacquer for food packaging (Agrimax EU-funded project [27] and TomaPaint S.r.l. [28]) have been proposed since 2014. However, to the best of the authors’ knowledge, high T_g_ polymer coatings from tomato peel waste were not a concern in previous studies [4,29,30,31]. A large fraction of industrial coatings is used on metals, and some of the larger end uses are automotive, appliance, container, and coil coatings. Among the different products available for metal protective coatings, epoxy resins based on bisphenol A (BPA) and epichlorohydrin are the most widely employed and have the highest market share of more than 90% [32]. Nevertheless, BPA is a harmful compound to both the environment and human beings. Indeed, it has been demonstrated that BPA can migrate to the human body, causing reproductive anomalies, cardiovascular disease, diabetes, and cancer [33,34,35,36].

The global BPA market is around 5.6 Mt/y [37], of which about 20% is used for epoxy resin production (i.e., 1 Mt/y), and cutin can be generated at a rate of the same order of magnitude [13,38]. In this scenario, cutin-based materials could perfectly meet the demand for replacing at least part of BPA-based resins, and their main component, 10,16-dihydroxyhexadecanoic acid (10,16-diHHDA), can be proposed as a building block for the development of fully biobased, sustainable coatings for metal protection.

The general objective of this work is therefore the development of high T_g_ and high cutin monomer content fully biobased coatings for general metal protection applications. A T_g_ higher than (at least) 45–50 °C is needed in order to obtain a coating surface with a sufficiently high scratch resistance. On the other hand, a cutin content preferentially higher than 25–30% by weight is advisable to maximize the exploitation of the particular renewable resource. The cutin-derived, dihydroxy hexadecanoic acid monomer was extracted from tomato waste peels and then formulated with other biorefinery monomers (i.e., glycerol, citric acid, succinic acid, and 2,5-furandicarboxylic acid) to develop crosslinked polyesters. Model coatings were prepared and characterized to assess their wettability, scratch hardness, chemical resistance, and adhesion to metal substrates, resulting in good physicochemical properties. Therefore, this work can pave the way for the use of cutin-based crosslinked polyesters as a green alternative to BPA-based resins for industrial coating applications.

## 2. Materials and Methods

### 2.1. Materials

Tomato pomace was kindly supplied by Tomato Farm S.r.l. (Tomato Farm S.r.l., Bettole di Pozzollo (AL), Italy). Each seasonal batch of biowaste (ca. 50 kg for each) was collected at two different times, during the first week of September 2022 and 2023. During the sampling, the canning plant was processing the following varieties of tomatoes: grape tomatoes, San Marzano tomatoes, which are a variety of plum tomatoes, vine tomatoes, and cherry tomatoes. All these tomato varieties were grown in fields located in Italy, mostly in northern Italy and a few in the south. All reagents and solvents were purchased from Merck (Merck Life Science S.p.A., Milan, Italy) and used without any further purification if not otherwise specified.

### 2.2. Synthesis Procedures

#### 2.2.1. Cutin Depolymerization and 10,16-Dihydroxyhexadecanoic Acid Isolation

In our previous work, we described a procedure for recovering 10,16-diHHDA from tomato-peel waste [39]. In this work, our protocol was further optimized in terms of both reaction time and yield of the reaction. Briefly, a known amount (around 30 g) of dried tomato peels is firstly degreased by Soxhlet employing n-hexane. The vegetable matrix is then depolymerized in alkaline conditions (ca. 1 M NaOH in methanol) under reflux for 3 h, filtered to remove solid residue, and left to rest overnight. Eventually, the crude solution is acidified by HCl down to ca. pH 3 to precipitate the desired 10,16-diHHDA compound that is recovered in the dichloromethane phase after extraction in a separatory funnel. In this work, the previously reported protocol was accurately followed, except for immediately processing the crude mixture resulting from the cutin depolymerization reaction. By immediately processing the crude mixture resulting from the cutin depolymerization reaction, a percentage increase of 50% in the yield of 10,16-diHHDA was achieved, when compared to the results obtained using the procedure developed in our previous work, without losing in terms of purity of the compound, as detailed explained in Section 3.1.

#### 2.2.2. Synthesis of Bis(2,3-Dihydroxypropyl) Furan-2,5-Dicarboxylate Prepolymer

A mixture of glycerol and 2,5-furandicarboxylic acid dimethyl ester (FDME) in a molar ratio of 2:1 was put in a three-neck round-bottom flask. The reaction was carried out at 200 °C for 4 h under an inert atmosphere and mechanical stirring, in the presence of a drop of Tin(II) 2-ethylhexanoate as a catalyst. A comparable reaction between 2,5-furan dicarboxylic acid diethyl ester and ethanol was carried out by Zhao et al., employing both conventional heating and microwave irradiation [40]. The time and temperature parameters applied in the current study for the reaction between glycerol and FDME were derived from the aforementioned research by Zhao et al. [40] Specifically, this synthesis was conducted under conventional heating conditions, adapting the time and temperature parameters to the reactants employed and the equipment.

#### 2.2.3. Polyester Resin Formulation and Preparation

The different crosslinked polyester compositions were prepared using the minimum volume of ethanol as a solvent and Ti(IV) isopropoxide (0.3 wt.%) as a catalyst. The mixture was stirred to achieve a homogeneous solution, then poured into a Petri dish and allowed to air-dry to evaporate part of the solvent. The resulting material was transferred into an oven and cured at 150 °C for 2 h at atmospheric pressure, and then kept under vacuum to complete the removal of volatile by-products. The polycondensation temperature employed in this study was derived from a previous paper on cutin-like co-polyester films [41]. Initially, a polycondensation time of 24 h was utilized in this study, as outlined in the abovementioned research. Subsequently, the duration was shortened, as this modification did not negatively affect the T_g_ and gel content values.

### 2.3. Design of Experiments (DoE)

To systematically explore the monomer composition effects on the coating’s T_g_, a design of experiments (DoE) approach was adopted. Specifically, the Box–Behnken design (BBD) and the response surface methodology (RSM) were employed. The experiments were established based on a BBD with three factors (i.e., (1) 10,16-diHHDA number of moles, (2) glycerol number of moles, and (3) OH/COOH molar ratio) and three levels (10,16-diHHDA content of 0.5–1.5 mol; glycerol content of 1–4 mol; OH/COOH molar ratio of 0.75–1.25) coded as −1, 0, and +1, as reported in Table 1.

Box–Behnken design is a rotatable quadratic design with no embedded factorial or fractional factorial points, where variable combinations represent points lying in the middle of the edges and the center of the variable space. The number of experiments (N) required for the development of a BBD is defined by the following equation:

(1) $$N=k^{2}+k+c_{p}$$

where k is the number of factors; and $c_{p}$ is the number of replicates of the central point [42].

In this case, it was chosen to consider three levels and one replicate of the central point, leading to 13 runs (Table 2). Furthermore, each experiment was performed in duplicate, resulting in a total number of 26 runs.

The experimental design enables the estimation of the system response at any experimental point within the investigation range [43]. The predicted response can be calculated using the response function, a regression equation in the following form:

(2) $$y=\beta_{0}+k\sum_{i=1}^{k}\beta_{i}x_{i}+\sum_{i=1}^{k}\beta_{ii}x_{i}^{2}+\sum_{j}\sum_{<i=2}^{k}\beta_{ij}x_{i}x_{j}$$

where y is the response; $x_{i}$ and $x_{j}$ are variables (i and j range from 1 to k); $\beta_{0}$ is the model intercept of coefficient; $\beta_{i}$, $\beta_{ii}$, and $\beta_{ij}$ are the interaction coefficients of linear, quadratic, and second-order terms, respectively; and k is the number of independent parameters (in this study, k=3) [44].

Minitab^®^ software (version 21.4.2.0, Minitab, Philadelphia, PA, USA) was used to explore the possibility of obtaining a material exhibiting a T_g_ value of at least 45–50 °C and with a sufficiently high cutin monomer content, starting from 10,16-diHHDA, glycerol, and succinic acid as reactants. This involved assessing the upper and lower limits of T_g_ as a function of 10,16-diHHDA content while minimizing the operational time of the analysis. To this end, T_g_ was set as the response of the experimental design, while gel content was considered as an internal validation parameter to ensure efficient crosslinking—and therefore good physicochemical properties—for the final products. Specifically, a minimum threshold of 98% gel content was set for polyesters to be considered suitable as metal protective coatings.

### 2.4. Coating Preparation Procedure

The final coatings were prepared by solubilizing all the monomers in ethanol at 70 °C, obtaining a homogeneous solution, which was then deposited on an A1008 steel substrate (Q PANEL, code S, 76 mm × 152 mm × 0.81 mm, ground finish, roughness = 0.51–1.14 µm, Q-Lab Corporation, Bolton, UK) using a K202 Control Coater (RK Print Coat Instruments Ltd., Royston, UK). Following the deposition, the coatings were cured at 150 °C for 2 h at atmospheric pressure and then kept under vacuum to complete the removal of volatile by-products.

### 2.5. Characterization Techniques

#### 2.5.1. Hydrogen Nuclear Magnetic Resonance (^1^H-NMR)

^1^H-NMR spectra were collected using a Bruker AV 400 MHz instrument (Bruker Corporation, Billerica, MA, USA. The samples were prepared by dissolving 1 mg of the sample in 1 mL of dimethyl sulfoxide-d6 (DMSO-d_6_).

#### 2.5.2. Differential Scanning Calorimetry (DSC)

DSC curves were collected using a Mettler-Toledo DSC 823e instrument (Mettler-Toledo, Columbus, OH, USA). The measurements were performed on 5–20 mg samples under a nitrogen flux. The thermal history included the following: (i) a first heating run from −50 °C to 150 °C (20 °C/min); (ii) a cooling run from 150 °C to −50 °C (20 °C/min); and (iii) a second heating run from −50 °C to 200 °C (20 °C/min). The glass transition temperature (T_g_) was determined as the inflection point of the second heating run.

#### 2.5.3. Gel Content Determination

Gel content measurements were performed by immersing each sample in 30 mL of ethanol and by maintaining it under magnetic stirring for 24 h at ambient temperature. Then, each sample was dried under vacuum for 24 h at 60 °C and then weighed. The gel fraction (${\%}_{gel}$) was calculated according to the following equation:

(3) $${\%}_{gel}=\frac{m_{f}}{m_{i}}\times 100$$

where $m_{f}$ is the mass of the sample after vacuum drying; and $m_{i}$ is the initial mass of the sample.

#### 2.5.4. Fourier-Transform Infrared (FTIR) Spectroscopy

FTIR spectra were collected using a Nicolet Nexus 760 FTIR spectrometer (Thermo Fisher Scientific, Waltham, MA, USA). The samples were prepared by dissolving the product in acetone and depositing a drop of the obtained solution on a KBr pellet. The measurements were performed at room temperature, in air, in transmission mode (64 scans at 4 cm^−1^ resolution), and in a range of 4000–1000 cm^−1^.

#### 2.5.5. Hydroxyl Number Determination

The hydroxyl number determination was performed by chemical titration following a standard procedure reported in the literature [45].

#### 2.5.6. Coating Characterization Tests

Thermogravimetric (TGA) analysis was performed employing a Q500 (TA Instruments, New Castel, DE, USA) instrument by heating from room temperature to 500 °C with a heating rate of 10 °C/min^−1^ in a nitrogen atmosphere.

Coating thickness was measured using a digital external micrometer, 0 ÷ 30 mm, MICROMASTER IP54 (TESA, Renens, Switzerland). The surface wettability of the coatings was determined at room temperature using an OCA 15Plus (DataPhysics Instruments GmbH, Filderstadt, Germany) instrument equipped with a CCD camera and a 500 μL Hamilton syringe, by measuring the static optical contact angle against ultrapure water. The scratch hardness of the coatings was assessed using dry samples through the Wolff–Wilborn method, using a set of 14 pencils (grades 9H to 9B), according to ASTM D3363-05 [46]. The chemical resistance of the coatings was evaluated through the solvent rub test, using methyl ethyl ketone (MEK) as the solvent, according to ASTM D4752 [47]. The adhesive strength of the coatings was estimated at room temperature using an ARW-T05 tester by measuring the pulling force required to detach a 20 mm diameter aluminum dolly adhered to the coating through an epoxy adhesive (EPX/DP460, cured at 25 °C for 24 h).

## 3. Results and Discussion

### 3.1. Cutin Depolymerization and 10,16-diHHDA Recovery

In order to assess the performance of cutin depolymerization and 10,16-diHHDA extraction, both the apparent yield ($\eta_{g}$) and the recovery yield ($\eta_{r}$) of the process were calculated. The former is defined as the ratio between recovered monomer mass and unprocessed tomato peel mass, while the latter is defined as the ratio between extracted monomer mass and the maximum amount of recoverable cutin from unprocessed tomato peel. The apparent and recovery yields were calculated according to the following equations:

(4) $$\eta_{g}=\frac{m_{cm}}{m_{bm}}\times 100$$

(5) $$\eta_{r}=\frac{m_{cm}}{{(m}_{bm}-m_{sr})}\times 100$$

where $\eta_{g}$ is the global yield; $\eta_{r}$ is the relative yield; $m_{cm}$ is the mass of recovered monomer obtained after the extraction; $m_{bm}$ is the mass of unprocessed tomato peel weighted before depolymerization; and $m_{sr}$ is the mass of solid residue obtained after depolymerization.

Starting from 30 g of unprocessed tomato peel and performing extraction 24 h after depolymerization [39], 8 ± 1 g of unreacted solid residue was obtained, resulting in average apparent and recovery yields of 10,16-diHHDA up to 30 and 40 wt.%, respectively. The variance in the quantity of the recovered final product is attributed to differences in biomass provenience and growth conditions, as well as to unquantifiable material losses along the single processing steps. A remarkable result was obtained by performing acidification and extraction immediately after depolymerization. In this case, it was possible to increase global and relative yields up to 45 and 60 wt.%, respectively.

At room temperature and atmospheric pressure, the isolated fatty acid appeared as a yellow-orange, waxy solid material. The ^1^H-NMR analysis demonstrated 96% purity of the extracted 10,16-diHHDA.

### 3.2. Cutin-Based Polyester Resins Formulation

A preliminary screening was performed to select the best candidates in terms of solvent and monomer mixture composition, to obtain a material exhibiting a T_g_ value of at least 45–50 °C and with a sufficiently high cutin monomer content.

To this end, solubility tests were performed, and all components showed better solubility in ethanol than in other common solvents tested, leading to the choice of using the former as a diluent in the following experiments. Then, citric acid and succinic acid were investigated as co-sources of –COOH groups as they are both Krebs cycle intermediates, making them easily available bio-based renewable raw materials suitable for food-contact applications. Eventually, the molar ratio between 10,16-diHHDA and glycerol was considered as a variable to study the effect of this parameter on the properties of the final product. Polyester resins were prepared starting from 0.5 g of 10,16-diHHDA, setting the molar ratio between –OH and –COOH groups equal to 1, and using ethanol as a solvent. T_g_ value and crosslinking efficiency were assessed using DSC and gel content measurements, respectively. The results obtained are reported in Table 3.

Succinic acid-based resins showed a slightly higher T_g_ and gel content than the citric acid-based ones. Looking at the ternary mixtures, it was concluded that by slightly increasing the glycerol content, the T_g_ of the material definitely increased as well. Nevertheless, the role played by the single components (as well as by the interactions among them) in determining the properties of the final product still needs to be extensively investigated.

**Figure 1:**
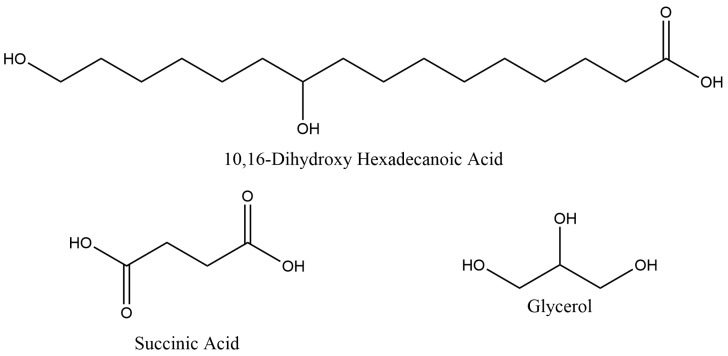
Chemical structures of the selected biobased monomers: 10,16-dihydroxyhexadecanoic acid, succinic acid, and glycerol.

To this end, a design of experiments (DoE) methodology was used to find the optimal composition for obtaining the most suitable material to be used as a coating in food applications. 10,16-diHHDA, succinic acid, and glycerol were selected as monomers (Figure 1).

### 3.3. Box–Behnken Design

To analyze the response surface design of the experiments, Minitab^®^ software (version 21.4.2.0) was employed. Specifically, collected data corresponding to the combinations identified in Table 2 were imported into the tool. This allowed the validation of the assumed significance of the main factors and formulation components through statistical evidence. To enhance accuracy, all experiments were performed in duplicate, following the principles of repetition and randomization. The functions “Create Response Surface Design” and “Analyze Response Surface Design” within the software were utilized.

The response function generated as the output of the analysis is given by the following regression equation:

(6) $$\begin{array}{ll} T_{g}= & -65.0486-4.16667x_{1}+2.72{222x}_{2}+169.0x_{3}-4.25x_{1}^{2}-0.361111x_{2}^{2}\\ & -81.0x_{3}^{2}+0.166667x_{1}x_{2}-7.00x_{1}x_{3}+5.00x_{2}x_{3} \end{array}$$

where $T_{g}$ is the glass transition temperature; and $x_{1}$, $x_{2}$, and $x_{3}$ are 10,16-diHHDA content, glycerol content, and OH/COOH molar ratio, respectively.

To evaluate the goodness-of-fit and the significance of the model, the determination coefficient ($R^{2}$), the correlation coefficient (R), and the adjusted determination coefficient ($R_{adj}^{2}$) were calculated, and the Analysis of Variance (ANOVA) was performed (Table 4).

**Figure 2:**
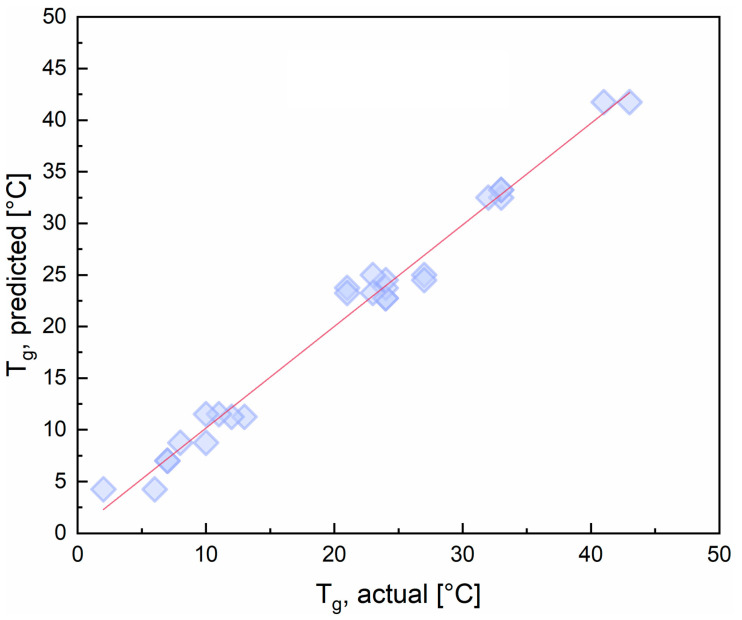
Parity plot for T_g_ with the linear fit for the data set.

The determination coefficient ($R^{2}$ = 0.985) indicates that 98.5% of the variation in the response is explained by the model. The adjusted determination coefficient ($R_{adj}^{2}$ = 0.975) is very high and close to the determination coefficient, confirming that the model was highly significant. Furthermore, the correlation coefficient (R = 0.992) is very high, indicating a good correlation between the actual and predicted responses. The statistical significance of the model is also confirmed by the very high F-value (F-value = 112.61) and the very low *p*-value (*p*-value << 0.05). The F-value and *p*-value are used to assess the null hypothesis for the regression, i.e., that the model does not explain any of the variation in the response. The higher than 1 the F-value and the lower than 0.05 the *p*-value, the stronger the evidence against the null hypothesis. Furthermore, the adequacy of the model can be validated in a visual way using a parity plot, displaying how accurate the estimated responses are against the experimentally observed ones (Figure 2). Each point has a pair of Cartesian coordinates (x,y) such that its actual and predicted T_g_ values represent its abscissa and ordinate, respectively. A good correlation between collected and estimated data is evidenced by the fact that the linear fit for the data points, namely the red line (y=0.984x+0.322, with $R^{2}=0.984$) plotted in the graph, is very close to a 45-degree line (y=x), representing the ideal case where predicted values match actual ones.

The regression equation was used to calculate the T_g_ values predicted by the response function and then compare them to the experimental results for all the samples. Furthermore, once the model was validated, it was used as a predictive tool to identify the cutin monomer content for a composition leading to the target T_g_ value of 50 °C (Simulation 1), as well as to estimate the glass transition temperature for the composition containing the highest 10,16-diHHDA weight fraction (Simulation 2). In both cases, the accuracy of the model was verified by preparing duplicates of the corresponding polyesters and characterizing them using DSC and gel content measurements. Run experiments and their corresponding collected data are reported in Table 5.

**Figure 3:**
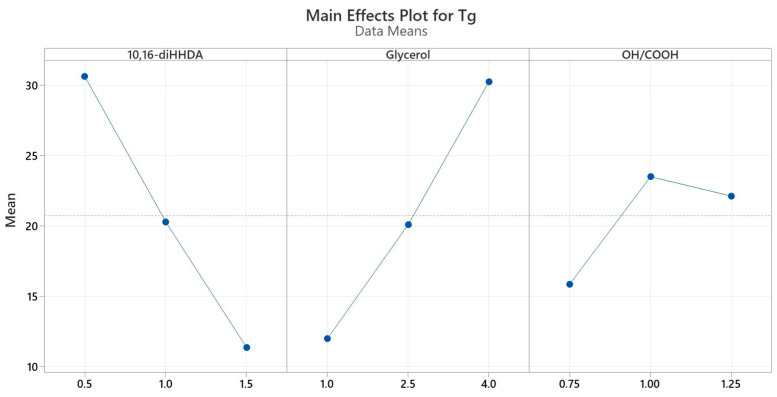
Main effects plot for T_g_, showing the mean for the T_g_ values computed for each level of the three factors, namely 10,16-diHHDA, Glycerol, and OH/COOH molar ratio.

As evident from Table 5, the Box–Behnken design demonstrated that there is no possibility of achieving the target T_g_ value of 50 °C within the investigation range defined in this study in terms of selected monomers (factors) and molar ratios between them (levels). As for the compositions identified by the BBD, the combination of 0.5 mol of 10,16-diHHDA, 4 mol of glycerol, and a stoichiometric OH/COOH molar ratio led to the highest glass transition temperature (i.e., 41–43 °C), but a too low cutin monomer content. The collected data outline a clear trend such that T_g_ is negatively affected by increasing 10,16-diHHDA and decreasing glycerol contents, as well as by OH/COOH molar ratios deviating from the ideal stoichiometric value (OH/COOH = 1). In the case of unitary value, the expectation is to achieve a closer-to-perfect network structure, free from defects and unreacted functional groups that could create defect points. To explore the effects of an off-stoichiometric value on the network structure, the OH/COOH molar ratio was selected as a factor, with its three levels (i.e., 0.75, 1, and 1.25), to assess its impact on glass transition temperature. The highlighted trend can be graphically visualized using the main effects plot, which displays the main effects of the analyzed factors on T_g_ (Figure 3).

Different levels of factors affect the response differently. The steeper the slope of the line, the greater the magnitude of the main effect. 10,16-diHHDA and glycerol monomers show constant influence on glass transition temperature in both the passages between levels −1 and 0 and between levels 0 and +1. Furthermore, their respective middle levels and means of T_g_ are almost coincident. A rather different behavior is underlined for the OH/COOH molar ratio, showing how glass transition temperature is way more dramatically affected by an excess of –COOH groups (OH/COOH = 0.75) than by an excess of –OH groups (OH/COOH = 1.25) in the system, since the slope of the line passing through levels –1 and 0 is steeper than the one passing through levels 0 and +1. In the case of an OH/COOH = 1.25, there were probably defects in the polymer networks induced by the excess of unreacted –OH groups that caused a slight decrease in the conversion degree of the crosslinking reaction—and therefore in the T_g_ value. Nevertheless, all of the compositions with an over-stoichiometric OH/COOH molar ratio showed a gel content greater than or equal to the threshold value of 98%, indicating a sufficient extent of crosslinking. Conversely, in the case of OH/COOH = 0.75, the excess of –COOH groups led to a surplus of mono- and bi-functional monomers and a lack of trifunctional monomers with –OH groups (i.e., glycerol). As a result, the system did not fulfill the following two conditions that generally maximize the extent of crosslinking: (1) a stoichiometric balance between reacting groups (in this case, –OH and –COOH), and (2) a mean functionality of the mixture higher than 2, favored by increasing polyfunctional reacting species and reducing monofunctional ones. Indeed, as reported in Table 5, all of the compositions with an under-stoichiometric OH/COOH molar ratio showed a gel content lower than the threshold value of 98%, which is undesirable for high-performance protective coatings. For the above-discussed remarks, polyesters obtained from the formulations with OH/COOH = 0.75 were considered unsuitable for the target application.

As for the model, the collected data demonstrate that the estimation is reliable not only within the study range defined in this Box–Behnken design but also outside. Indeed, the accuracy of the prediction was verified for both Simulation 1 (entry 1 in Table 5) and Simulation 2 (last entry in Table 5)—the latter being a combination of levels encompassed in the BBD; the former presenting a glycerol content of 6 mol, which is higher than 4 mol, corresponding to the glycerol level coded as “+1”. In both cases, the experimentally observed T_g_ values were fairly consistent with the ones calculated using the regression equation generated as the output of the analysis. Therefore, the model is a valid and useful predictive tool to estimate glass transition temperature for any combination of 10,16-diHHDA, glycerol, and succinic acid monomers that is represented by a point falling within the investigation range and/or reasonably close to its boundaries.

Considering this statistical analysis, the variation in the initial formulation can represent a good strategy to modify the T_g_ of the final coating, adapting it to the desired requirements. As already stated in Section 3.2, a minimum T_g_ value of at least 45–50 °C was fixed, because this enabled a good trade-off between sufficiently high scratch hardness, commonly associated with high T_g_ values, and good post-cure formability of the already coated metal strips without cracks and defects, usually achieved with lower T_g_. In any case, the highest value of the T_g_ obtained with this statistical analysis is around 40 °C, which is still lower than the target value of 50 °C. On the other hand, any attempt to further increase the T_g_ value—even by considering different levels rather than the ones defined in this Box–Behnken design (e.g., a glycerol content of 6 mol, as in Simulation 1)—would result in a material containing a too low 10,16-diHHDA weight fraction, preventing a proper valorization of such an abundant agri-food waste as tomato peels.

To increase the T_g_ and optimize the 10,16-diHHDA content in the final coating, it was decided to introduce a stiffer heterocyclic biobased prepolymer in place of the aliphatic, highly mobile glycerol, i.e., the bis(2,3-dihydroxypropyl) furan-2,5-dicarboxylate monomer (Section 3.4). Thanks to the presence of a furan ring, this compound can form strong intermolecular interactions due to its ring conformational constraints, leading to a reduction in the mobility of the polymer chains in crosslinked coatings. This can cause a decrease in the free volume in the crosslinked polymeric network, increasing its glass transition temperature. Furthermore, this prepolymer is a derivative of 2,5-furandicarboxylic acid (FDCA), one of the “hottest” biobased monomers in modern green chemistry, available from various integrated biorefinery routes, and a candidate to replace fossil-based terephthalic acid in many polymer-related applications [48].

### 3.4. Synthesis of the Bis(2,3-Dihydroxypropyl) Furan-2,5-Dicarboxylate Prepolymer

**Figure 4:**
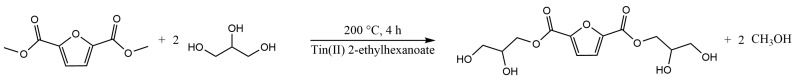
Simplified reaction scheme of the transesterification between 2,5-furandicarboxylic acid dimethyl ester and glycerol, yielding bis(2,3-dihydroxypropyl) furan-2,5-dicarboxylate as the main product and methanol as polycondensation by-product.

To further increase the T_g_ of the final product, the base formulation of the cutin-based resins was therefore modified by replacing glycerol with an FDCA prepolymer constituted by the bis(2,3-dihydroxypropyl) furan-2,5-dicarboxylate. A simplified reaction scheme for the synthesized prepolymer is shown in Figure 4.

**Figure 5:**
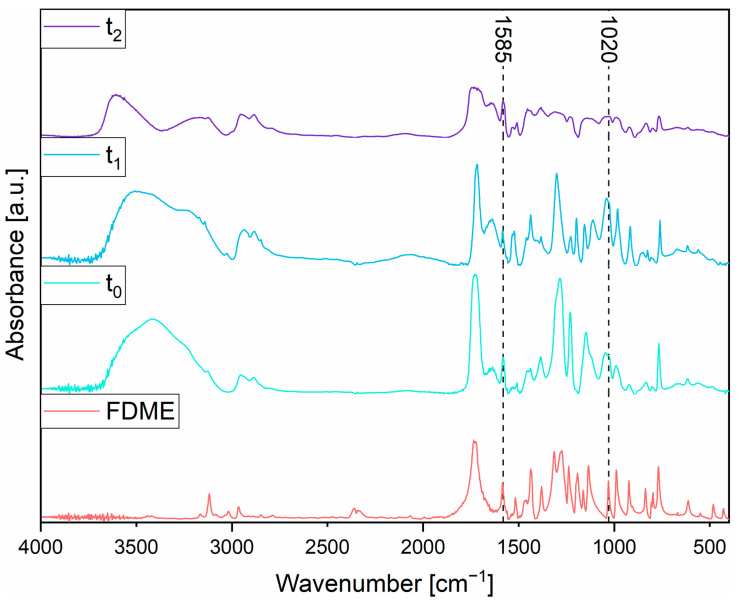
FT-IR spectra: 2,5-furandicarboxylic acid dimethyl ester (FDME); the system at the beginning of the reaction (t_0_); the system after two hours since the beginning of the reaction (t_1_); and the system after four hours (t_2_).

The reaction was run in a stoichiometric excess of glycerol (1 mole of FDME to 2 moles of glycerol). The progress of the reaction was monitored by periodically sampling the crude mixture and measuring FT-IR spectra (Figure 5).

The signal at 1585 cm^−1^, attributed to the stretching vibration of the C=C bonds in the furan ring, was used as an invariant reference peak to normalize all the spectra. The broad absorption band in the region 3700–3100 cm^−1^ is ascribed to the stretching vibration of terminal O–H groups in the glycerol and the final FDCA prepolymer. The peaks detected at 3131 cm^−1^ are associated with the stretching vibration of the =C–H bonds in the furan ring. The absorption band in the region 3000–2840 cm^−1^ is related to the stretching vibration of –CH_2_ and C–H in the backbone. The signal at 1735 cm^–1^ is attributed to the stretching vibration of the C=O bond in ester groups. The broad absorption band in the region 1310–1200 cm^−1^ is ascribed to the stretching vibration of the C–O–C bond in ester groups. The peak detected at 1020 cm^–1^ is associated with the stretching vibration of the C–O–C bond in the furan ring.

The progress of the reaction can be assessed by looking at the broad adsorption band in the region 3700–3200 cm^−1^. As the reactants are converted into the products, methanol is released and evaporated because of the operating conditions (the reaction was run at 200 °C, and methanol boils at 65 °C). The decrease in the total number of –OH groups within the system caused by the polycondensation led to a decrease in the intensity of the adsorption band related to the stretching vibration of terminal O–H groups. Another interesting marker of the condensation reaction is the splitting of the unique, unstructured very broad signal into two well-distinct bands at around 3700–3400 and 3400–3100 cm^−1^, starting from the unique broader band.

**Figure 6:**
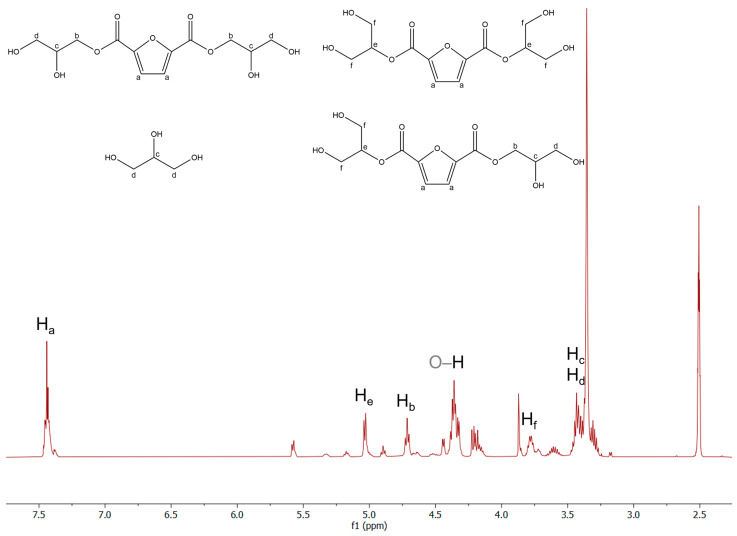
^1^H-NMR spectrum in DMSO-d_6_ of the synthesized FDCA prepolymer.

^1^H-NMR was also performed to check the prepolymer structure (Figure 6).

The peak detected at 7.44 ppm is attributed to the two ideally homotopic H_a_ atoms of the furan ring. Due to the different isomers available for the condensation reaction of FDCA, the signal is not a singlet but a more complex multiplet. The doublet centered at 5.03 ppm and the triplet centered at 4.72 ppm are ascribed to H_e_ and H_b_ atoms, respectively. The complex signal in the region 4.41–4.29 ppm is associated with the H atoms of the terminal hydroxyl groups in glycerol and the co-products. The multiplet in the region of 3.80–3.75 ppm is attributed to H_f_ atoms. The complex signal centered at 3.43 ppm is ascribed to H_c_ and H_d_ atoms in glycerol and its co-products. Except for the characteristic residual not-deuterated solvent signal, centered at 2.50 ppm, namely dimethyl sulfoxide-d_6_ (DMSO-d_6_), the unassigned signals are likely attributed to some oligomers formed during the reaction.

The equivalent hydroxyl number of the synthesized prepolymer was experimentally determined in view of its use to replace glycerol and supply each system with the same number of –OH groups provided in the glycerol-based polyester resins. To this end, the following equation was used:

(7) $$m_{prepolymer,i}=\frac{n_{glycerol,i}\times 3}{{OH}_{exp}}$$

where $m_{prepolymer,i}$ is the mass content of the FDCA prepolymer needed in the *i^th^* formulation; $n_{glycerol,i}$ is the molar content of glycerol in the *i^th^* formulation; and ${OH}_{exp}$ is the experimentally determined equivalent hydroxyl number of the synthesized prepolymer.

To assess the possible beneficial effects of the higher rigidity of the central furan ring on glass transition temperature, two combinations of levels encompassed in the study range defined by this BBD were selected and modified. Specifically, glycerol was replaced with the FDCA prepolymer in the formulations exhibiting the highest and lowest 10,16-diHHDA content, namely, compositions coded as “Simulation 2” (last entry in Table 5) and “3” (entry 3 in Table 2), respectively. As demonstrated by the results reported in Table 6, the introduction of the prepolymer into the reaction system led to a remarkable increment of around 40 °C in the glass transition temperature of the final crosslinked coatings when compared to previously obtained materials. Based on this evidence, two additional formulations showing a T_g_ around 10–15 °C and a gel content not lower than 98% were modified as well, expecting the same increment of around 40 °C in the glass transition temperature of the final crosslinked coatings as in the previous cases, as a consequence of replacing glycerol with the FDCA prepolymer. The compositions coded as “11” (entry 11 in Table 2) and “8” (entry 8 in Table 5) were selected to prepare model polyester coatings. These concerning data are reported in Table 6.

**Figure A1:**
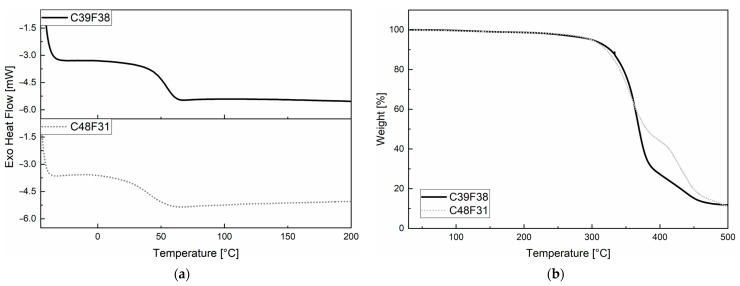
DSC thermograms (**a**) and thermogravimetric analyses (**b**) for the two selected coatings, named as “C39F38” (constituted by 39 wt.% of 10,16-diHHDA, 38 wt.% of FDCA prepolymer, and 23 wt.% of succinic acid) and “C48F31” (constituted by 48 wt.% of 10,16-diHHDA, 31 wt.% of FDCA prepolymer, and 21 wt.% of succinic acid), in a nitrogen atmosphere.

The composition constituted by 39 wt.% of 10,16-diHHDA, 38 wt.% of FDCA prepolymer, and 23 wt.% of succinic acid (entry 2 in Table 6, from here on referred to as “C39F38”) was selected as the most promising candidate for coating applications. Indeed, it exhibited the best trade-off between glass transition temperature and 10,16-diHHDA content, meeting the requirements for both ensuring good physicochemical properties of the final material and quantitative valorization of tomato-peel waste. To assess the potential effects of a 10,16-diHHDA-enriched formulation on the chemical, mechanical, optical, and physical properties of the coating, the composition constituted by 48 wt.% of 10,16-diHHDA, 31 wt.% of FDCA prepolymer, and 21 wt.% of succinic acid (entry 1 in Table 6, from here on referred to as “C48F31”) was investigated as well, resulting in a T_g_ lower than 50 °C, set as a threshold. DSC thermograms and thermogravimetric analyses for C39F38 and C48F31 coatings are shown in Figure A1. The two cutin- and FDCA-based crosslinked polyesters showed less than 2% weight loss at 200 °C during TGA in an N_2_ atmosphere, indicating good thermal stability even at high temperatures. Both coatings were also characterized by assessing their wettability, scratch hardness, chemical resistance, and adhesion to the substrate (Table 7).

The chemical resistance of the cutin-based polyester coatings was evaluated by a solvent rub test using methyl ethyl ketone (MEK) as the solvent. Both materials successfully passed more than 100 double rubs without failure or breakthrough of the surface, demonstrating high chemical inertness. An adhesive strength higher than 2 MPa for the prepared films on steel substrates was estimated by performing pull-off adhesive tests, confirming their potential applicability as the inner lining of food and beverage cans. As a result, even a percentage of around 50 wt.% of 10,16-diHHDA does not negatively impact the functionality of the material in terms of the properties here investigated by technological tests.

## 4. Conclusions

In this paper, we showed that the monomer derived by the depolymerization of tomato’s cutin present in the peels of this fruit (i.e., a biowaste from the canning industry) can be converted into fully biobased crosslinked networks by a high-temperature poly-esterification reaction with other biorefinery co-monomers bearing hydroxyl and carboxylic groups such as succinic acid, glycerol, and 2,5-furandicarboxylic acid. To investigate the effect of the co-monomer contents on the T_g_ of cutin-derived coatings, a Box–Behnken design and response surface methodology were utilized for coatings comprising 10,16-diHHDA, glycerol, and succinic acid. Among the compositions identified by the Bex–Behnken design, a combination of 0.5 mol of 10,16-diHHDA, 4 mol of glycerol, and a stoichiometric OH/COOH molar ratio led to a higher glass transition temperature (i.e., 41–43 °C) but a cutin monomer content lower than desired. To obtain a coating with a T_g_ higher than 45–50 °C and a cutin monomer content exceeding 25–30 wt.% to exploit the agro-waste renewable sources, a furan dicarboxylic acid-based prepolymer was developed and used as a substitute for glycerol. Through careful optimization of the monomer mix composition, this study showed how coating films characterized by remarkable properties determined from technological tests, like chemical resistance and good adhesion to metals, as well as relatively high levels of scratch hardness and T_g_ values of around 50 °C, can be obtained. The employed formulations completely avoid the use of fossil-based and toxic external curing agents like polyisocyanates and melamines. The “green” profile and the technological properties of cutin-based polymer films make them potential candidates as internal can coatings for the food industry, a market that is nowadays still dominated by BPA-based epoxy coatings. While the results obtained in this study from technological characterizations are promising, it is important to note that further research is essential to comprehensively assess the potential of cutin-based metal protective coatings. This includes an in-depth examination of their anticorrosion properties, toxicological profiles, durability, and post-cure metal formability through rigorous testing.

## Figures and Tables

**Table 1:** Three factors and the corresponding three levels were selected for the Box–Behnken design.

Levels	Factors		
	10,16-diHHDA [mol]	Glycerol [mol]	OH/COOH Molar Ratio
−1	0.5	1	0.75
0	1	2.5	1
+1	1.5	4	1.25

**Table 2:** Uncoded and coded Box–Behnken design for the three-factor system used in this study.

Std. Order	10,16-diHHDA [mol]	Glycerol [mol]	OH/COOH Molar Ratio
1	0.5 (−1)	1 (−1)	1 (0)
2	1.5 (+1)	1 (−1)	1 (0)
3	0.5 (−1)	4 (+1)	1 (0)
4	1.5 (+1)	4 (+1)	1 (0)
5	0.5 (−1)	2.5 (0)	0.75 (−1)
6	1.5 (+1)	2.5 (0)	0.75 (−1)
7	0.5 (−1)	2.5 (0)	1.25 (+1)
8	1.5 (+1)	2.5 (0)	1.25 (+1)
9	1 (0)	1 (−1)	0.75 (−1)
10	1 (0)	4 (+1)	0.75 (−1)
11	1 (0)	1 (−1)	1.25 (+1)
12	1 (0)	4 (+1)	1.25 (+1)
13	1 (0)	2.5 (0)	1 (0)

**Table 3:** Glass transition temperature (T_g_) and gel content values for coatings were obtained by mixing different percentages of 10,16-dihydroxyhexadecanoic acid (10,16-diHHDA), citric acid (CA), succinic acid (SA), and glycerol (Gly) in the preliminary screening. The molar ratio between –OH and –COOH groups was kept equal to 1 for all the mixtures considered.

Coating Components	Weight Fractions [%]	Molar Fractions [%]	T_g_ [°C]	Gel Content [%]
10,16-diHHDA	75	67	−11	92
CA	25	33		
10,16-diHHDA	83	67	−7	94
SA	17	33		
10,16-diHHDA	38	19	33	98
SA	43	52		
Gly	18	29		
10,16-diHHDA	33	15	35	99
SA	47	54		
Gly	21	31		
10,16-diHHDA	25	11	43	98
SA	51	56		
Gly	24	33		

**Table 4:** Analysis of Variance (ANOVA) and Model Summary of the quadratic regression model for the prediction of glass transition temperature (T_g_).

Source	DF	Adj SS	Adj MS	F-Value	*p*-Value
Model	9	3135.62	348.40	112.61	0.000
R = 0.992		$R^{2}$ = 0.985		$R_{adj}^{2}$ = 0.975	

**Table 5:** Box–Behnken design and simulation experiments with the actual and predicted responses (“T_g_, actual” and “T_g_, predicted”, respectively) and 10,16-dihydroxyhexadecanoic acid weight fraction, sorted by glass transition temperature from the highest to the lowest.

Run No.	10,16-diHHDA [mol]	Glycerol [mol]	OH/COOH Molar Ratio	T_g_, Actual [°C]	T_g_, Predicted [°C]	10,16-diHHDA Weight Fraction [%]
Simulation 1 ^a^	0.5	6	1	48	50.13	8
				47		
16	0.5	4	1	43	41.75	12
3	0.5	4	1	41	41.75	12
7	0.5	2.5	1.25	33	32.50	19
12	1	4	1.25	33	33.25	23
25	1	4	1.25	33	33.25	23
20	0.5	2.5	1.25	32	32.50	19
13	1	2.5	1	27	25.00	32
5 ^b^	0.5	2.5	0.75	27	24.50	14
4	1.5	4	1	24	25.00	27
14	0.5	1	1	24	24.50	33
17	1.5	4	1	24	22.75	27
18 ^b^	0.5	2.5	0.75	24	24.50	14
26	1	2.5	1	23	25.00	32
10 ^b^	1	4	0.75	23	23.25	17
1	0.5	1	1	21	23.75	33
23 ^b^	1	4	0.75	21	23.25	17
11	1	1	1.25	13	11.25	52
24	1	1	1.25	12	11.25	52
8	1.5	2.5	1.25	11	11.50	40
21	1.5	2.5	1.25	10	11.50	40
9 ^b^	1	1	0.75	10	8.75	41
22 ^b^	1	1	0.75	8	8.75	41
19 ^b^	1.5	2.5	0.75	7	7.00	31
6 ^b^	1.5	2.5	0.75	7	7.00	31
15	1.5	1	1	6	4.25	55
2	1.5	1	1	2	4.25	55
Simulation 2	1.5	1	1.25	5	−0.44	60
				1		

^a^ This point falls outside the investigation range defined in this Box–Behnken design. ^b^ This composition showed a gel content lower than 98%.

**Table 6:** Glass transition temperature (T_g_) and gel content for coatings were obtained by mixing different weight percentages of 10,16-dihydroxyhexadecanoic acid (10,16-diHHDA), the prepolymer synthesized from 2,5-furandicarboxylic acid dimethyl ester (FDME) and glycerol (FDCA prepolymer), and succinic acid (SA).

Resin Components	Weight Fractions [%]	–OH Fraction from 10,16-diHHDA [%]	–COOH Fraction from 10,16-diHHDA [%]	T_g_ [°C]	Gel Content [%]
10,16-diHHDA	48	50	31	41	100
FDCA prepolymer	31				
SA	21				
10,16-diHHDA	39	40	25	54	100
FDCA prepolymer	38				
SA	23				
10,16-diHHDA	28	29	18	53	100
FDCA prepolymer	45				
SA	27				
10,16-diHHDA	7	8	4	83	100
FDCA prepolymer	56				
SA	37				

**Table 7:** Key technological features of selected coatings.

Coating Components	Weight Fractions [%]	Thickness [µm]	Water Contact Angle [°]	Pencil Hardness	MEK Test [Double Rubs]	Pull-Off Strength [MPa]
10,16-diHHDA	39	42	101 ± 1	HB	>100	>2.15
FDCA prepolymer	38					
SA	23					
10,16-diHHDA	48	44	100 ± 1	HB	>100	>2.20
FDCA prepolymer	31					
SA	21					

## Data Availability

Publicly available datasets analyzed in this study can be found here: https://polimi365-my.sharepoint.com/:f:/g/personal/10542832_polimi_it/EqB6cZ0lcV1FvQQbzn6_IqsBTKhEJ-OClJ43JBOML0xveQ?e=jfw4j1, accessed on 24 February 2024.
